# Order Parameter in Bacterial Biofilm Adaptive Response

**DOI:** 10.3389/fmicb.2018.01721

**Published:** 2018-07-26

**Authors:** Kumar Selvarajoo

**Affiliations:** Biotransformation Innovation Platform (BioTrans), Agency for Science, Technology and Research A^*^STAR, Singapore, Singapore

**Keywords:** biofilms, criticality, systems biology, *Bacillus subtilis*, emergent properties, *Escherichia coli*

## Maintext

Bacteria are free living organisms (disordered system), with no correlation in their single cell behaviors (Elowitz et al., 2002). When exposed to environmental or pathogenic threats, they are able to aggregate, often within a self-secreted extracellular polymeric substances (EPS) matrix, resulting in a highly correlated ordered structure called biofilm (Flemming et al., 2016), with the latter being strongly associated with cell-to-cell communication referred to as “quorum sensing” (Papenfort and Bassler, 2016). The formation of biofilms has been an intense area of research as they are resistant to diverse environmental perturbations or carefully targeted treatment strategies (Koo et al., 2017).

To understand the complex behavior of bacteria cooperative behavior, numerous computational and spatial measurement techniques have been developed and analyzed, see reviews by Pérez-Velázquez et al. (2016), Dunham et al. (2017), and Bridier et al. (2011). In a recent paper in this journal, Gingichashvili et al. (2017) have taken a computational modeling and imaging strategy to understand *Bacillus subtilis* biofilm development. They first performed *in vitro* experiments on GFP-tagged *B. subtilis* over 4 days in two different growth media. Using the imaging data, they, subsequently, developed a computerized model that converted the image into frequency and amplitude signal variables. The model next helped to (i) measure the rate of *B. subtilis* biofilm growth, (ii) track temporal and spatial bacterial GFP production and, (iii) identify key morphological differences between mature and under-developed biofilms. Another computational approach that could utilize the imaging data to infer self-organizing rules is the cellular automata, a discrete methodology that utilizes user defined simple rules to predict the behavior of an automaton or cell in time, space, and state (Wolfram, 1983; Tang and Valocchi, 2013).

Although, undoubtedly, these approaches or methodologies have made interesting progress to the biofilm modeling research, nevertheless, I do feel that more fundamental theoretical approaches are required to understand the mechanistic basis for biofilm evolution.

There is ongoing debate on whether physical laws do exist in biology. Both theorists and biologists are proposing that physical or chemical laws cannot be directly brought into biology, but a novel theory for organisms is needed (Soto et al., 2016). Although I do agree that theories for biology require rethinking, I cannot but help to quote several important discoveries in physics that are also present in biology (Jeong et al., 2000; Wilsenach et al., 2017). In particular, scale invariance leading to fractal structure and power laws are nowadays ubiquitously observed from biological data (Piras and Selvarajoo, 2015; Simeoni et al., 2015). Non-linear approaches, such as chaos theory, fall into an important research domain that can be explored to investigate complex self-organizing behavior of biofilm. Chen-Charpentier and Stanescu (2011) have used stochastic modeling together with a chaos method to predict biofilm growth on medical implants. However, the difficulty facing the neat predictability of the models is the general lack of obtaining reliable parameter values. As such, we see very limited contributions from chaos theory to-date.

One way to overcome the parameter dependence is to look for global (as opposed to local) properties. Introduced in 1987, self-organized criticality (SOC) is observed as a general property of dynamical systems, whose macroscopic (or average) behavior shows a critical point leading to a phase transition (Bak et al., 1987). In other words, a dynamic system is able to evolve spontaneously toward a critical point, without fine tuning system parameters, for a phase transition. The transition of water (free moving molecules) to ice (fixed lattice) at freezing point is a well-known example. Such transformation will leave the system invariant, or in a collective mode. At the critical point, the system achieves universality, that is, all differences between individuals will be reduced to follow common “universal” properties. To distinguish the phases, order parameter, such as network activity, can be investigated (Hesse and Gross, 2014). Do living systems display SOC?

The onset of bacterial biofilm development demonstrates scale invariance in biology. In line with this theme, a recent study investigated the mechanisms for *Escherichia coli* biofilm dynamics when exposed to lytic T7 phages (Vidakovic et al., 2018). Biofilm of varying ages, grown in microfluidic flow chambers, were continuously exposed to phages for 12 h. A phase transition was observed when biofilms were killed by phage if they are grown for <60 h, else they were collectively protected from phage-mediated death. Subsequent tests on the shift to stationary phase and genetic mutations were ruled negative. To find the mechanism for the phase transition, they investigated the EPS of the *E. coli* biofilm, making single-gene knockout mutants of proteinaceous curli fibers, flagellar filaments, cellulose, poly-β-1,6-N-acetyl-D-glucosamine, colanic acid, and type 1 fimbriae. Each mutant was still able to retain their 3-D biofilm structure. Among these, the curli fibers mutant was most susceptible to phage-mediated killing. Further investigations revealed that curli were produced at the upper regions of biofilms between 48 and 60 h after biofilm growth, and curli fibers localized in the space between cells. Single cell fluorescence imaging showed biofilms lacking curli fibers allowed the diffusion of phages into “pores” between cells created in the mutants. Although diffusion through the space alone is not the only mechanism for phage-killing, Vidakovic et al. showed that the cell to cell alignment is the crucial order parameter that prevents phage attacks. That is, amyloid curli fibers are the key single EPS component that reorganizes the internal structure of the biofilm so as to prevent the penetration of T7 phages. Thus, in this case, measuring the growth of curli fibers can act as an order parameter.

In my opinion, this is a remarkable demonstration in two ways; (i) it shows a living system, such as *E. coli*, displays key physical properties such as SOC (leading to phase transition that allows the species to survive under threats), and (ii) it achieves them through rather surprisingly simple mechanisms, such as preventing of diffusion of the pathogen into the community.

Now that we can safely establish that order parameter is observable from experiments, it will be interesting to explore, in the future, the transcriptome-wide global response of biofilm formation and progress. Although there are works that have already investigated transcriptomics of biofilm regulators, an example is *Saccharomyces cerevisiae* (Cromie et al., 2017), such works do not deal with investigating the global order parameter. We recommend the use of information-theory based statistics to infer the local and global character of biofilm response (Tsuchiya et al., 2009; Giuliani et al., 2018; Selvarajoo et al., 2018). The local response are attributes of highly changing small number of variables, whereas, the global response characterizes a wide-range of lowly changing variables. For example, one could check whether the global molecular expression correlation increases during the ordered response of biofilm formation, as would be anticipated in the context of the universality principal of dynamic systems.

## Author contributions

The author confirms being the sole contributor of this work and approved it for publication.

### Conflict of interest statement

The author declares that the research was conducted in the absence of any commercial or financial relationships that could be construed as a potential conflict of interest.
